# Elaborating a systems methodology for cascading climate change impacts and implications

**DOI:** 10.1016/j.mex.2020.100893

**Published:** 2020-04-19

**Authors:** Nicholas A. Cradock-Henry, Justin Connolly, Paula Blackett, Judy Lawrence

**Affiliations:** a Manaaki Whenua – Landcare Research, Lincoln, New Zealand; b Deliberate Consulting, Hamilton, New Zealand; c National Institute of Water and Atmosphere, Hamilton, New Zealand; d Victoria University of Wellington, Wellington, New Zealand

**Keywords:** Climate change adaptation, Vulnerability, Systems thinking, Feedback, Complex problems

## Abstract

New research is drawing attention to the potential for climate change to generate cascading impacts and implications across linked human-environment systems, requiring closer accounting of these interactions to anticipate the emergence of surprises and feedbacks. However, there is little practical guidance for those interested in characterising, identifying or assessing cascades, and few empirical examples. In this paper, we elaborate a systems-based methodology to identify and evaluate cascading climate change impacts and implications. We illustrate its application using the case of a participatory process with urban infrastructure managers, facing the legacy effects of damaging earthquakes and the prospect of future climate change. The results show the proposed approach and visualisation of cascades as causal diagrams provides a robust and flexible analytical framework. The use of systems thinking, visual aids, interactive discussion and expert elicitation generated valuable information about potential cascades, their interactions across domains of interest, and the implications for management. The process can provide a basis for further empirical application and advance methodological and conceptual development.

*Specifically, the systems methodology:*

• Identifies interdependencies and interconnections which may serve as transmission pathways for climate-related impacts;
• Enhanced stakeholders’ understanding of multiple causes and effects of climate change; and
• Produced a useful visual aid for stakeholders to explore cascading impacts and implications, and opportunities for intervention.

**Table:** Specifications table

Subject Area:	Environmental Science Natural resource management
More specific subject area:	*Climate change impacts*
Method name:	*Systems methodology for cascading climate change impacts and implications*
Name and reference of original method:	*Lawrence J, Blackett P, Cradock-Henry NA. Cascading Climate Change Impacts and Implications. Climate Risk Management. CLRM_2019_163*
Resource availability:	*N/A*

**Method details**

## Background

There is increasing evidence of the effects of climate change on linked human-environment, or social-ecological systems [5,^6^,^15^,16]. The consequences of higher mean temperatures, increasing climate variability and more frequent extremes, are evidenced in the dramatic rise in weather-related losses over the last decade, placing additional pressure on already marginalised systems, communities and locations [14,^19^,^40^,51]. Furthermore, the felt impacts of climate change do not happen in isolation [43]. While increased rainfall can result, for example, in higher rates of soil erosion, increased flooding and landslides, they can have implications across and between multiple domains [31,32]. The interaction and feedback between local environments (such as steep hillsides), downstream receiving catchments and tributaries, and flood protection measures, are only just beginning to be recognised in the literature [39]. At a global level, Anthropocene risk – emerging from the interactions between multiple processes – has implications for management, and requires cross-scale interventions [30].

The potential for climate change impacts and implications to ‘cascade’ across and between multiple domains, is prompting new research aimed at conceptualising and empirically examining their combined effects in diverse settings [9,^18^,^23^,^32^,42]. While the cascade effect has been studied elsewhere – most notably in disaster risk research [2,3], and studies of networked infrastructure and lifelines [17,^20^,29] – there are few empirical examples from climate change research, and limited practical guidance for researchers, policy makers and practitioners in how cascades might be identified, characterised and considered as part of planning and management.

The ‘Cascading Climate Change Impacts and Implications’ (CCCII) project in Aotearoa-New Zealand (New Zealand) was one of the first studies to develop and apply a conceptual and methodological approach to cascades in the context of climate change [34], [58]). In it, we explore the implications of cascades to help focus adaptation interventions and enhance resilience. In this paper, we extend the exploration of cascades in the context of climate change and elaborate on the five-step process used that is based on the theory and practice of systems thinking. Systems thinking is a scientific methodology and set of tools that deal with complexity, ambiguity and mental models [27,35]. Importantly, systems thinking focuses on the whole system as well as the constituent parts and their interactions. It therefore provides a framework for managing change and complexity, through the understanding of dynamic feedback embedded in complex systems [27].

In the climate change context, systems thinking allows decision makers to anticipate the long-term consequences of their decisions and actions, by considering the interdependencies between them, as well as the unintended consequences of policies and strategies [4]. As such it can support comprehensive and robust adaptation planning, reducing the risk of maladaptation and a narrow focus on a single problem [57]. Overall, systems thinking provides a collective understanding of how a systems functions and a common language for diverse stakeholders to enable deep dialogue and consensus building [35,^37^,46].

## Method details

In the literature on climate change impacts assessment, a number of different methods have been advanced in recent years, from probabilistic, model-based scenarios of future change, to in-depth participatory assessments based on rich qualitative insights from stakeholders. Increasingly however, there is a call for more integrated approaches to impacts assessment that combine both top-down and bottom-up perspectives, to gain insight into the diverse contexts for adaptation. Many of these integrated approaches use elements of systems thinking [10,^46^,55] applying it to a wide range of topics related to climate change impacts, implications and adaptation [21,^26^,^32^,^36^,45]. By providing a formalised and accepted language for articulating relationships, synergies and trade-offs – systems-based approaches enable integrative and holistic assessments, incorporating multiple dimensions of sustainability [21,^41^,^46^,56]. Furthermore, there is increasing evidence for a ‘participatory’ turn in impacts assessment, and greater emphasis on both scientific and lay knowledge, to enhance the relevance, credibility and legitimacy of assessments to improve the likelihood of having a positive effect on desired outcomes [7,^8^,^14^,^22^,^44^,46].

Cascading climate change impacts and implications are inherently complex and contested problems [9,32], with significant uncertainties and evolving complexities. As such, it is ill-suited to investigation using the traditional scientific method which relies on systematic experimentation which can be validated – and its credibility enhanced – through repetition [8]. For complex problems such as this, there are multiple stakeholders involved, with diverse perspectives and assumptions, undertaking different actions towards possible solutions [48], requiring a different approach. To overcome the difficulties of establishing the credibility of learning outcomes from this type of enquiry, it has been suggested that the method of enquiry and area of concern, should be declared in advance, creating an intellectual structure to reflect upon research outcomes [11,^24^,48]. Clearly stating theoretical ideas, research themes and a clear statement of the methods makes it easier for others to recover the research process and evaluate findings [11]. Without it, results of investigation may be perceived as anecdotal accounts of an interesting case, limiting opportunities to comparison and sharing [1,11].

We use system dynamics as a “declared in advance” methodology [11]. The process as outlined here, is intended to enable others to critique the process, and further apply and refine it, to meet the criteria for recoverability [11]. The process is informed by established systems-based methodologies and group model building to gain insight into the cascading impacts and implications of climate change. The steps we followed were used to focus dialogue in stakeholder workshops on how different climate change risks (e.g. extreme rainfall events) resulted in impacts that were transmitted to other sectors, activities or domains [34].

While the choice of methods may vary, assessments typically include problem identification, system description (and any normative outcomes desired), assessment of impacts, identification of options, followed by implementation and evaluation. For this project we developed a simplified impacts assessment focused on cascades using a methodology that combined systems thinking with a participatory approach, grounded in stakeholders’ understandings and perceptions, to characterise cascading climate change impacts and implications [34]. We used a five-step process as follows:

1. Problem definition
2. Mapping critical infrastructure
3. Generate narratives of cascading impacts and implications
4. Develop system map
5. Evaluate and refine

The stepwise framework is aimed at generating evidence to enhance understanding of the scale and scope of cascading climate change impacts, particularly with a focus on critical local infrastructure. The application of the framework can be used to identify key interdependencies, feedbacks and co-dependencies, and determine how impacts and implications might extend across multiple sectors.

### Problem definition

The first step in the process was to articulate the problem and better understand stakeholders’ perceptions of the issue. In systems thinking, boundaries are flexible and can be set at a scale that is appropriate to the question of interest. In this case, boundaries were drawn to include factors that influenced the function of infrastructure, where infrastructure was broadly defined as three waters (wastewater, stormwater, and water supply services provided by local government in New Zealand), flood and inundation protection structures, utilities, and road networks.

Stakeholder workshops were used to develop a shared view of the system of interest – including the interaction of roles and responsibilities, norms and the political and decision-making context. A simple scenario of changes in key climate change variables (i.e. precipitation, temperature, and frequency and variability of extremes and how emergent impacts propagate) was used to prompt initial discussions exploring the impacts of climate change on a selected domain (e.g. urban water infrastructure). Structured questions and activities solicited information about the issue and affected systems.

Questions used for defining the problem included:

• What is the system of concern? (set the boundaries of the system)
• How would you describe the current system?
• What are the effects of climate change on the system and its management?
  ○ *slowly emerging impacts*, e.g. sea level rise, plant and animal diseases
  ○ *widening climate variability*, e.g. drought, increased flood frequency
  ○ *extremes*, e.g. coastal storm surge, intense rainfall, wind
• What are the implications for decision making inside and outside the system?

The resulting information was used to develop a problem statement and a ‘rich picture’ of the current situation and of stakeholders’ views of the problem [38,50]. Problem statements should typically include both the problem context and an agreed version of problem to be addressed. Our problem statement is as follows:

Local government decision-makers would benefit from having the ability to understand the cascading impacts of climate on critical infrastructure. Ideally this would help decision-makers understand how these impacts would flow on to other elements of the various complex socio-ecological systems that such critical infrastructure supports.

### Mapping critical infrastructure

This step was used to establish a conceptual and empirical basis for system model development. At its most fundamental level an infrastructure system performs a variety of functions for society, including services to the public, often without them even realising, that enable society to operate to an expected level. Workshop dialogue was used in conjunction with the CIrcle Tool (Critical Infrastructures: Relations and Consequences for Life and Environment) – an interactive mapping tool – to identify services that may be affected by climate change, and their interdependencies [25].

**Fig. 1:**
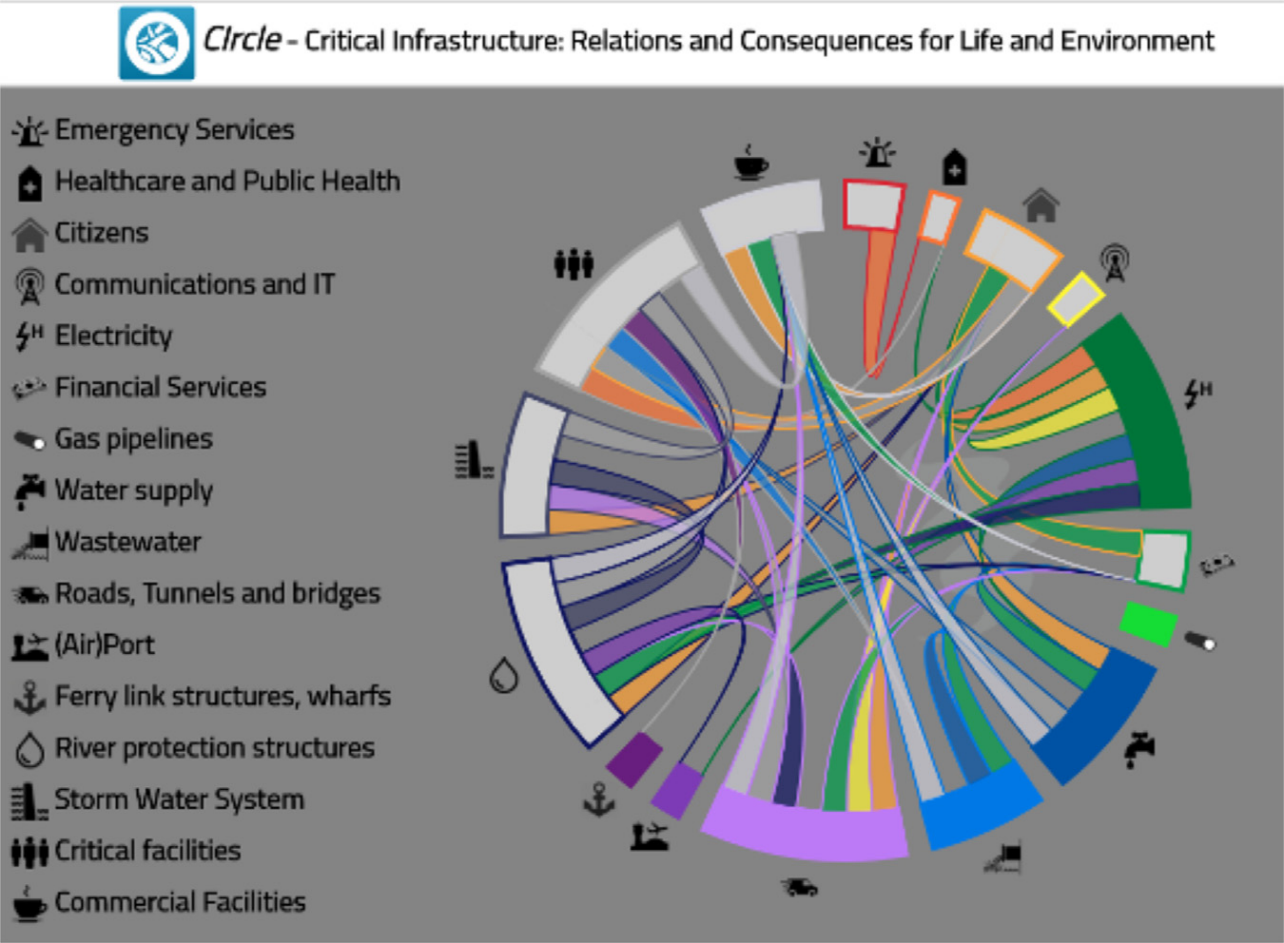
(Appx. here) Example of CIrcle derived dependencies between impact categories described by workshop participants.

Participants identified critical infrastructure within the geographical area of interest and considered both how climate change (i.e. slowing emerging impacts, widening climate variability and extremes) might affect that infrastructure and how the different infrastructure types were connected with each other, and with other social and economic components of the system. Components included groundwater, governance, water supply, financial services, healthcare and more (Fig. 1).

Workshop discussions were used to identify the number of times each input category interconnected with another in the system. These critical nodes indicated potential priority areas for decision makers’ attention, and helped establish critical dependencies with widespread consequences. For example, rising sea levels and/or more extreme rainfall events are interconnected to outcomes in other parts of the system. The resulting floods, for example, may impede road access and contaminate streams. Health and community well-being are also affected by damp in homes, and limiting access to social infrastructure such as schools and hospitals [34].

The aim was to conceptualize activities relevant to the problem situation. The output was used to inform the building blocks of potential cascades and a system map by describing how parts of the system were understood to be connected. Visually displaying the connections between infrastructure components in real time, during the workshop, provided an opportunity to iterate system representation, and discuss amongst each other, the significance of interdependencies, helping stakeholders understand the complex and interdependent relations between critical infrastructure systems.

### Narratives of cascading impacts and implications

In other studies, using system dynamics and development of systems diagrams, often begins with the use of simple, familiar models (e.g. maps, visualisations, tables and lists). For this step, information from workshop participants, media stories about impacts of recent storm events and the authors’ previous research on climate change impacts and implications [33] was used to develop narratives of cascading impacts [34].

Narratives are used here to refer to plausible storylines of cascading impacts and implications. Stories are increasingly used to communicate information on climate change, and can provide important tools to support decision-making and strategic planning [13,28]. The narratives elaborated on cascading systems loops, using locally-relevant examples, in some cases informed by recent events. Using examples described in media, such as flood events or other disruptions, enhanced the relevance of narratives and helped decision makers consider implications of cascades, which might otherwise be framed as discrete impacts in place and time.

The workshop participants provided information about the variables, interconnections and feedbacks for each domain of interest (e.g. for urban water infrastructure, flood protection and lifelines infrastructure). Maps were used to focus dialogue on the local context, and information was abstracted to develop accessible narratives to inform a generalised system model to better describe interactions.

### Develop systems map

The underlying presumption in systems dynamics is of inter-connectedness and the natural integration of biophysical, physical, cultural, social, political, organisational, and economic elements that enable co-construction of systems to explore and explain what is observed in the real world. The relationships between the factors in the system have a direction: ‘A is related to B’ to, for example, ‘as A increases B decreases’, which enables identification of reinforcing or balancing loops within the system. Accordingly, vicious or virtuous cycles can be exposed (reinforcing loops) as well as where relationships have a ‘cancelling-out’ effect (balancing loops). For additional detail and discussion of systems maps, see Supplementary Materials.

**Fig. 2:**
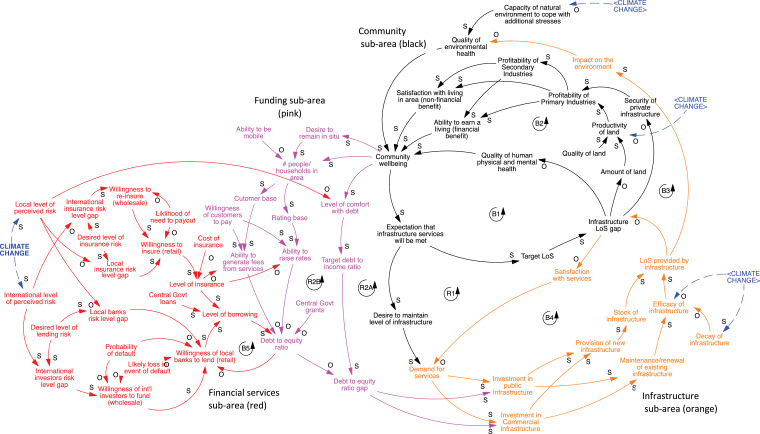
(Appx. here) Systems map showing cascading impacts across multiple domains.

To further explore these dynamics, we used Vensim modelling software (Ventanna Systems, Inc.) to create a systems map that represented the causal relationships described by participant stakeholders (Fig. 2). The systems map (also known as a causal loop diagram, since it shows dependencies and interdependencies) shows how the different types of climate change impacts (e.g., extreme events and slowly emerging impacts) have similar interdependencies, feedback loops, and generate similar cascades across other domains. This allows for the consideration of these characteristics at a more generic conceptual scale. The systems map highlights relations, or causal links, between infrastructure systems, and can be used to analyse and visualize cascading impacts and implications elsewhere. Individual cascades relating to particular infrastructure were aggregated into a single system map, because it was evident that strong commonality existed between how the individual systems operated. As a result, the map represents all types of infrastructure for all the climate impacts considered.

Once the system was described and drawn, we considered how it may be affected by climate change (i.e., slowly emerging, widening climate variability, or extreme events) or specific policy intervention to address those impacts – where interventions are deliberate actions to achieve a desired change(s). The implications of changes or interventions were traced through the system and examined for any unintended consequences. Potential cascades were then discussed and recorded.

Initially two versions of the systems map were developed: one high-level, containing 13 factors; and a second detailed one, containing 59 factors (4 times more complex based on the number of factors). Participants were more comfortable with the detailed version of the map (Fig. 2).

### Evaluate and refine

Once constructed, the systems map was tested with a sub-set of key informants in a small workshop and in one-on-one meetings. Structured dialogue was used to step through the systems map, focusing first on the four sub-systems (infrastructure, community, and funding and financial services) and then the causal relations between them [58]. The aim was to ensure the systems map captured their perspective of how the system functioned. Providing an opportunity for reflection on the systems map, and discussing how stakeholders might usefully use it in their own work, also contributed to enhancing the relevance and legitimacy of findings [8]. Minor amendments were made based on stakeholder feedback, and a final system map was produced.

The systems maps shows complex and interdependent cascades linked by seven feedback loops across four sub-areas of the system. Importantly, the systems map illustrates how the cascades arise from a climate change impact and move across space and organizations, affecting ecological, social, and economic domains. The cascades can move through a connected social-ecological system, combining or exacerbating the impacts, because of external policy decisions or concurrent impacts or events. While the three climate-derived impacts of concern (high-intensity rainfall events, sea-level rise and drought) can be characterised for the purposes of building cascades, it is important to remember that the different types of impacts can occur simultaneously at the same geographical location. This could create multiple simultaneous cascades that recombine in potentially unpredictable ways or accelerate movement towards a threshold. The incidence of surprises cannot be characterised by definition but cannot be ruled out as further stressors on the systems.

The seven feedback loops and dependencies are the critical elements of the systems mapping process. The systems map presented here (Fig. 2) is a summary of the overall system of four sub-areas of the systems (community – black; infrastructure – orange; funding – purple; financial risk – red) and factors driving the cascades in each domain.

## Method validation

The participatory process, which involved multiple stakeholders with diverse perspectives derived from their disciplinary and functional responsibilities in each of the domains, was able to motivate insights into the complexity of interdependencies, and enabled joint learning [34]. This and other examples of group model building [54] with policy-makers and managers have been shown to enhance understanding of system dynamics, causal relations and the effects of decisions in other contexts [12,^47^,[52], [53], [54]].

Workshop participants described the use of systems dynamics and model development as highly beneficial for thinking about the wider implications of climate change impacts. In particular, more subjective links and causal relationships (e.g. *Community wellbeing; Perceived level of risk* (in relation to climate impacts); *Willingness to insure)*, that are not included in probabilistic or economic models were incorporated in the systems map, providing a richer picture of the complexities of the system. As such, the systems map was effective at making multiple interconnections and interdependencies within the system, explicit, demonstrating the emergence of the cascading phenomena [34]. For example; the circular nature of the relationship between community wellbeing and infrastructure was insightful, as was the observation that increasing levels of infrastructure will, over time, gradually increase the expectations for even greater levels of infrastructure – continually adding upward pressure to community expectations. Another important insight was the significant influence of finance and insurance on the ability to invest in new or for maintaining existing infrastructure.

The systems map also demonstrated the *multiple* pathways of causality that climate change impacts have on other variables within the wider system. This highlighted that a changing climate will affect the system at several points and may combine or compound the impacts, because of external policy decisions or concurrent impacts or events.

The explicit labelling of feedback loops in the system map also provided insights on the presence of balancing or reinforcing loops. While the majority of the map is made up of balancing loops, several potentially vicious loops were identified. One example of this is the reinforcing loops relating to infrastructure funding – either through rates (taxes) or direct payment for services. When financial and community wellbeing are high, there is a corresponding capacity to rate or pay for infrastructure, positively reinforcing improved financial and community wellbeing. If the performance or level of infrastructure is sufficiently disrupted though, this loop could ‘flip’ into a negatively loop – with low financial and community wellbeing resulting in a reduced ability to rate or pay for infrastructure. The presence of reinforcing loops also provided a means to consider the governance implications of actions within the system [49], as well as any unintended consequences. Systems mapping is agnostic to organisational boundaries (internal or external) and the open discussion provided a forum for participants to better understand of how actions in one governance domain may impact (or be impacted by) other domains.

A key tension when applying systems thinking to a particular context is between too much, or too little detail to apply when generating the variables. Domain experts such as policymakers and managers, typically have detailed knowledge about their area of responsibility, but in this case, there was a need to consider multiple domains, requiring significant aggregation. Ultimately, the level of detail presented in the final map will be determined by how comfortable the participants are that their system domain is sufficiently represented to enable exploration and deliberation.

## Conclusions and future research directions

This paper has described an application of system mapping (based on System Dynamics causal-loop diagramming) to explore the cascading impacts of climate change. A mixed methods approach was used, drawing on group model building using workshops and interviews, to elicit insights on the impacts of climate change from a range of participants with functions and experience in urban infrastructure and asset management, flood risk management, financial services. This information was collated by researchers into a systems map that synthesised the inter-relationships among the domains. This provided insights on how the impacts of climate change in one domain could cascade into other domains and how domains were interdependent creating circular (and repeating) cascades.

The systems methodology elicited interdependencies and interconnections (especially with finance and insurance) and it enabled an understanding of *multiple* causes and effects. Furthermore, it enabled exploration of the influence of different scales; exposure of *vicious cycles* in the system; and investigation and understanding of the *governance* implications of domain impacts and actions, within and between systems [34].

More detailed applications of this methodology in an area nested within the wider system would add to the knowledge of particular domains. For example, further exploration of the impact of perceived climate risk within the finance and insurance industries and its potential impact on the provision of infrastructure would contribute to investments in long-lived assets on which communities rely. The impact that this methodology had on the mental models of workshop participants remains untested and could be explored further. Finally, future research could also develop small-scale simulation models of the dynamics described in these (or any future maps), to test the narratives shown in the systems map for their ability to reflect the interactions and their implications in a changing climate.

## Declaration of Competing Interest

The authors declare that they have no known competing financial interests or personal relationships that could have appeared to influence the work reported in this paper.

## Appendix Supplementary materials

Image, application 1
