# Supplementary material for: Elaborating a systems methodology for cascading climate change impacts and implications
Source: MethodsX. 2020 Apr 19;7:100893. doi: 10.1016/j.mex.2020.100893 (PMC7184528; doi:10.1016/j.mex.2020.100893)
Supplement: Supplementary file 1 [file mmc1.docx]

**S1. SYSTEM DYNAMICS AND CLIMATE CHANGE**

The world that we live in is a highly interconnected place of causality and effect (Bollinger et al., 2014; Galaz et al., 2011; Keys et al., 2019; Liu et al., 2013), and many of the problems relating to long-term sustainability are increasingly value-laden and characterised by complexity (Dentoni et al., 2018; Moser et al., 2012). In order to contribute to a solutions-oriented science of sustainability, there have been calls for greater emphasis on integrated, systems-oriented approaches, tools and processes. Systems thinking focuses on the dynamic interrelationships of different elements shaping complex sustainability issues. It takes a systemic view of sustainability issues rather than breaking them down into a series of discrete elements that can be addressed separately, and focuses on the causes – rather than proximate symptoms – of undesirable behaviour or patterns being experienced in our natural environment (Abson et al., 2017; Agrawal et al., 2013; Frame, 2008).

Systems thinking is a conceptual framework and set of tools that have been developed to help make these patterns of interconnectedness clearer (Senge, 2006). The term is used often interchangeably with the academic discipline of ‘System Dynamics’. Systems’ tools help us understand the structure of a set of various interacting factors that present in a behaviour we are trying to understand. This helps us better understand which parts of a system are having the most influence on a certain behaviour and allow us to identify areas of leverage to influence outcomes (Fischer and Riechers, 2019; Meadows, 2010; Sterman, 2000). In this section, we briefly review some of the relevant tools used in systems dynamics, followed by their application in a process for characterising climate change cascades.

***System dynamics***

System Dynamics seeks to understand the structure and behaviour of complex systems and find appropriate policies to tackle particular problems (Vennix et al., 1996). Proponents of System Dynamics describe it as a holistic approach that has the potential to bridge academic disciplines, as well as the gap between science, policy/management organisations, and the public (Costanza et al., 1998). As a result, it is often presented as a potential means to explore, and potentially resolve, complex, multi-stakeholder, multi-domain, trans-disciplinary problems, like those regularly encountered in environmental debates (Williams et al., 2017), such as climate change (Ison, 2010; Ross et al., 2015). A system-dynamics framing can enable dependencies and interdependencies to be identified and, thus, provides a framing for consideration of cascading climate change impacts.

*System structure*

System Dynamics has several unique properties which distinguish it from other approaches and make it useful for obtaining insights into both how cross domain, multi-stakeholder problems function and where interventions might occur (Holling, 2001; Ison, 2010; Williams et al., 2017). Central to understanding the dynamics of a particular system of interest, is the ‘system structure’. The system structure includes three core elements: accumulation; interconnections; and feedback loops (Forrester, 1968; Sterman, 2000). These three elements provide the building blocks for mapping systems, and include the key variables (or factors) and the relationships they have one with the other (van den Belt, 2004), that can be used to demonstrate various characteristics of complex behaviour such as the capacity to adapt or transform (Barnes et al., 2017; Folke et al., 2010); self-organisation (Sterman, 2000) and emergence (Williams et al., 2017). We briefly review each element in turn.

### *Accumulation*

Accumulation (or decumulation) is used in system dynamics to describe the way(s) in which things ‘build up’ (Sterman, 2000). Accumulation and decumulation can be used to articulate a range of issues under examination, from supply chain storage to company morale, to greenhouse gas emissions in the atmosphere or inequality in society and can be illustrated using the bathtub analogy (Figure 1).


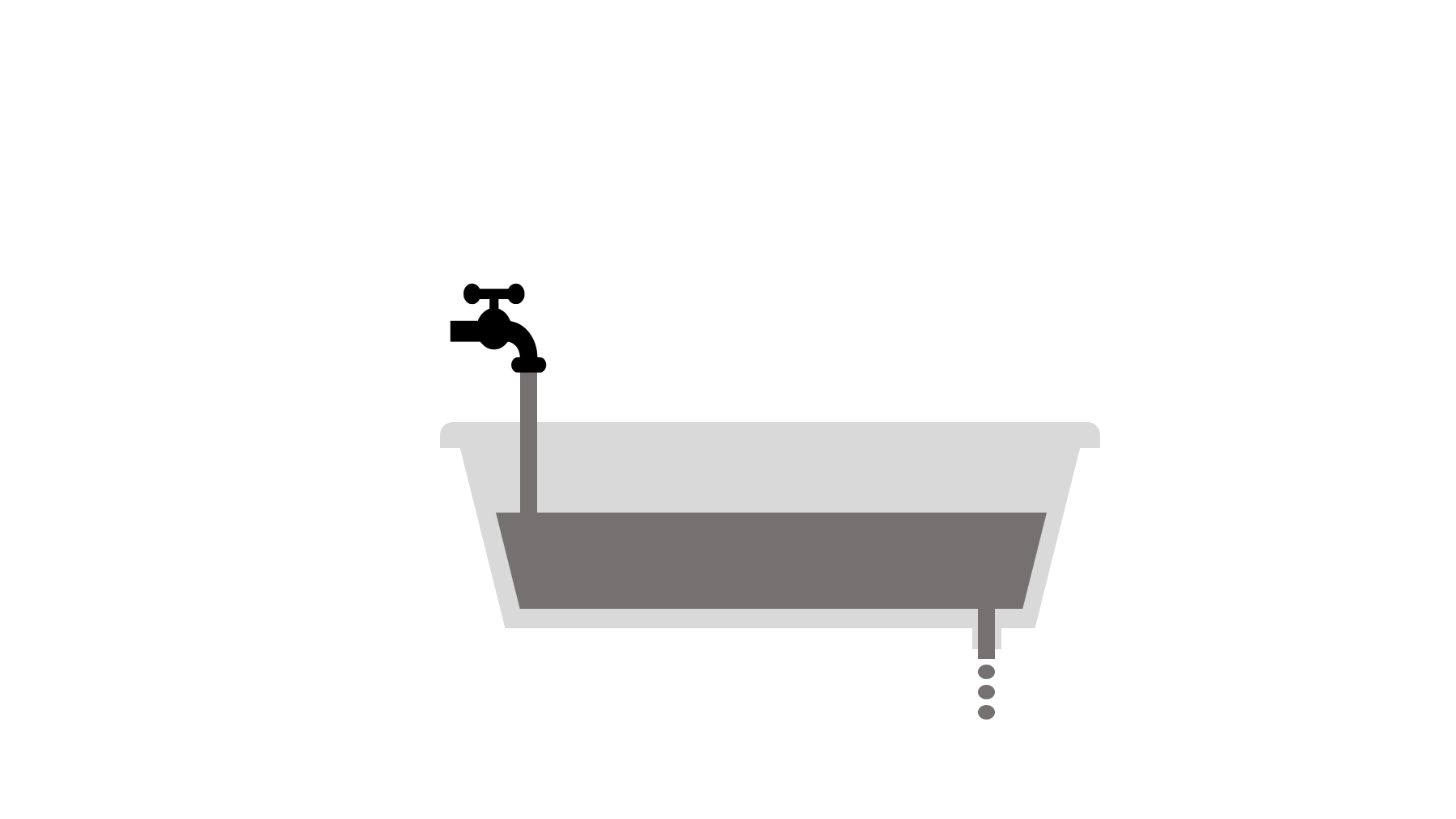


**Figure 1. The bathtub analogy - used to help understand where things ‘build up’ or ‘decline’ in a system**

Accumulation (or decumulation) is usually captured in a systems map, in which variables are named – using nouns or noun phrases – and articulated in such a way that they have a sense of direction (i.e. they can change up or down). Both quantitative and qualitative variables can be represented in system maps, depending on the purpose of the research, data available and the nature of the problem under study. Factors/variables represent the relevant elements of any given problem, at the selected scale, irrespective of disciplinary divisions (i.e., if the variable is biological, economic or social). This has considerable merit where complex problems overlap traditional disciplinary boundaries or different domains. Furthermore, qualitative directions within the name should be avoided (e.g. instead of ‘low debt’ use ‘debt’), yet if it is not possible to avoid inherent directionality within a name, use the positive version (e.g. ‘happiness’ instead of ‘unhappiness’) (Sterman, 2000).

The behaviour of these factors (the level of water in the bath) is influenced by the many interconnections between causal factors related to it, and whether any of those are connected in a chain of circular causality or feedback loops.

### *Interconnections and feedback loops*

Interconnections in system maps represent causal relationships between factors and should represent the direction of change that one factor has on another. Where factors/variables are connected in a chain of circular causality - either directly or mediated through other factors or variables - feedback loops are established.

Feedback loops seek to move the understanding of problems and issues from being independent events separated in time and space (linear causality), to the current state of connected variables continually influencing each other (circular causality) (Forrester, 1968; Sterman, 2000). Such loops either maintain balance or reinforce certain activity (Figure 2). If they are reinforcing, this can create a change in the magnitude of the other variable(s) in a way either a positive (virtuous cycle) or negative (vicious cycle) way (Sterman, 2000).

The argument is that understanding feedback loops is essential to successful interventions (or intended changes to the system). Linear descriptions of systems that do not account for feedback loops risk misrepresenting the system and resulting in interventions which generate unintended consequences or unexpected outcomes. In some cases, the impacts may be in another domain or a different sector.


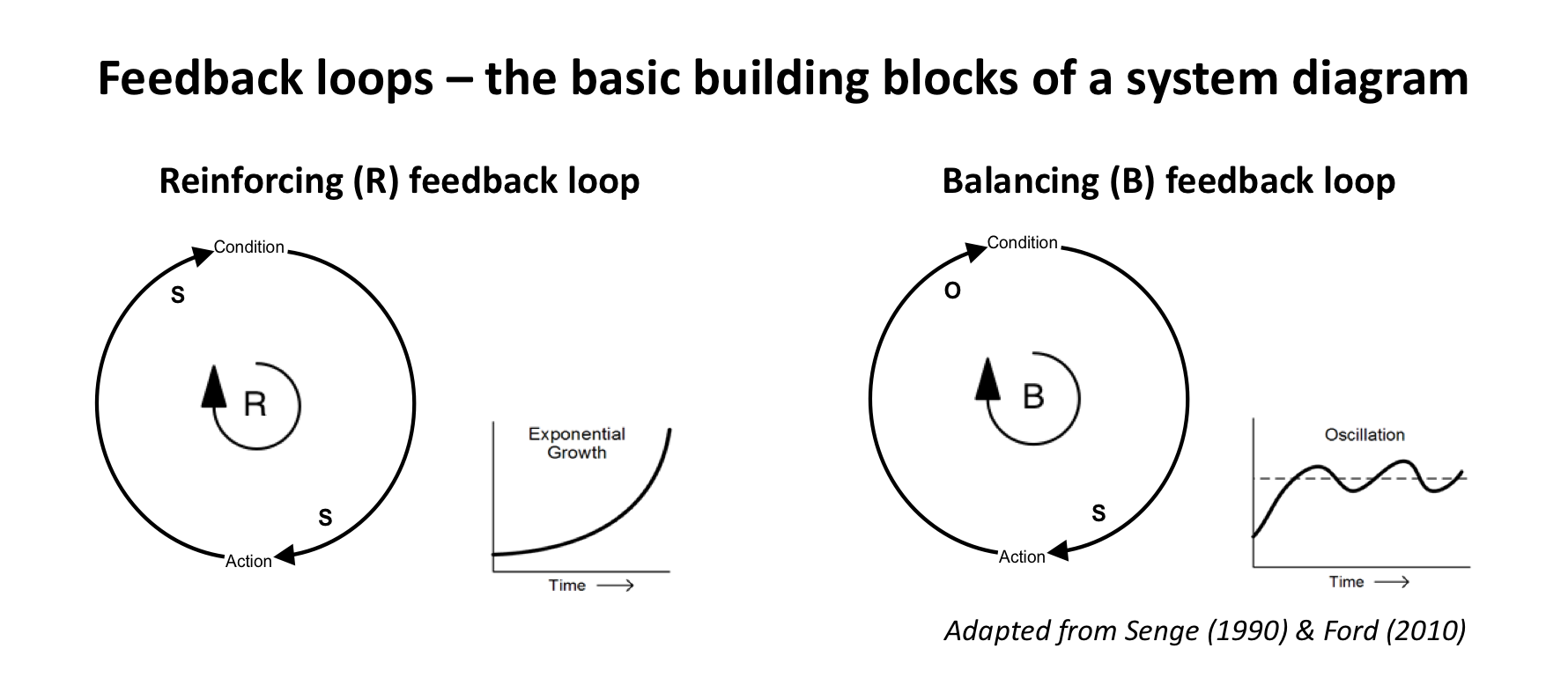


| In a ***reinforcing feedback loop***, the influence from any factor will transfer around the loop and influence back on the originating factor in the *same* direction. This has the effect of *reinforcing* the direction of the original influence, and any change will build upon itself and amplify (in either a positive or negative direction). | In a ***balancing feedback loop*,** the influence from any factor will transfer around the loop and influence back on the originating factor in the *opposite* direction. This has the effect of *balancing or cancelling out* the direction of the original influence. |
| --- | --- |

**Figure 2: The two types of feedback loops - balancing and reinforcing**

The arrows between factors represents how a change in one factor *causes* *directional change* in another - that is, if A goes up or down, then B goes up or down. If both factors move in the same direction the arrow is labelled as a ‘same’ relationship (with an ‘s’). If the factors move in different or opposite directions the arrow is labelled as an ‘opposite’ relationship (with an ‘o’ (Figure 3). Same and opposite have been used here, whilst they are also sometimes known as ‘positive’ (same) and ‘negative’ (opposite) relationships.

Any distinguishable *delay* between a change in one factor and its impact on another is annotated as a *double line crossing the arrow*.

Identifying multiple loops provides useful insight to a system, yet not all feedback loops are created equal; different loops can dominate the dynamics of a system at different times. One useful piece of system structure that can help to identify this is the ‘goal/gap’ structure. In the goal/gap structure there is a factor or node that combines both a desired (‘goal’) level of something, with an actual (reality) level of something. The difference between the goal and actual levels is the ‘gap’. The size of this ‘gap’ influences the strength of the causal influence that this gap has on other factors. Generally, the greater the gap, the stronger that loop will operate to reduce that gap.

A simple example of a ‘goal/gap’ structure in operation is shown in Figure 5: filling a glass of water. Rather than being a linear process, this is a circular process. When the glass is empty the actual level (an empty glass) is a long way from the ‘desired’ level (a full glass). Therefore, in response, the tap is opened a long way to allow a strong flow of water to make up this shortfall. As the level of water in the glass nears the ‘goal’ the openness of the tap is likely to be reduced (you don’t want it to overflow!), decreasing the loop’s strength. This reduces the *strength* of the water flow (the dominance of the loop) until eventually it is stopped altogether when it reaches the ‘goal’ (the desired level of water) and the tap is closed. Mapping out such relationships in a multiple-loop system can identify where the influences of one loop may have an impact on other loops. Goal/gaps feature prominently in the system map developed to describe the cascading climate change impacts and implications in the companion paper (Lawrence et al., 2020) (see also Figure 5).


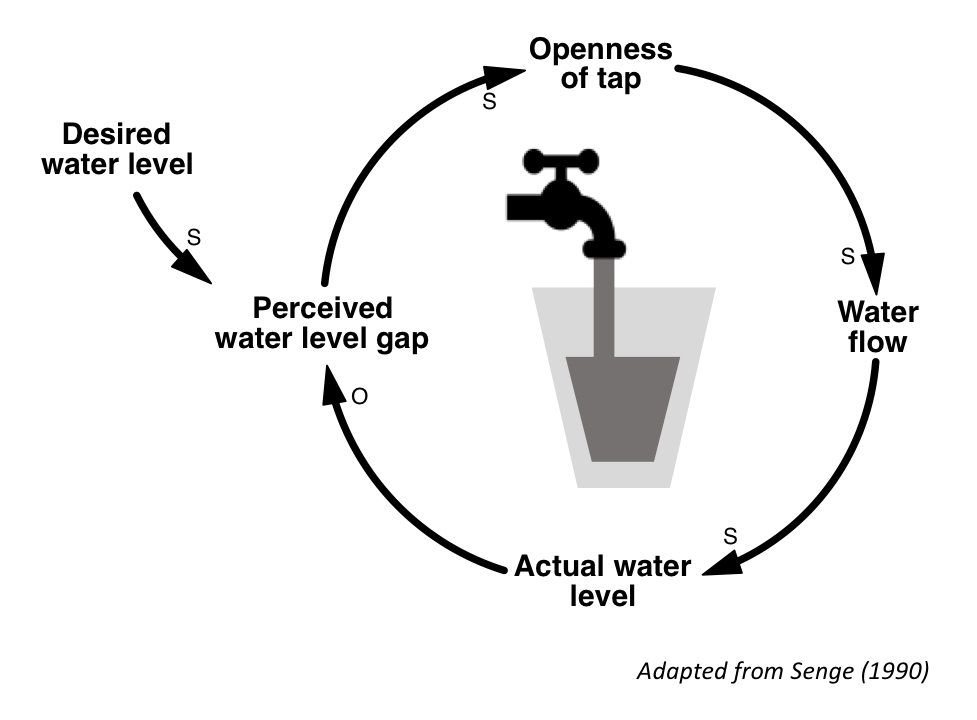


**Figure 3: Example of a ‘goal/gap’ structure in a system map**

Understanding such feedbacks is essential for successful interventions (or intended changes to the system) (Sterman, 2000). Linear descriptions of systems that do not account for feedback loops run the risk of misrepresenting the system, resulting in interventions that generate unintended consequences or unexpected outcomes. In some cases, the impacts may be in another domain or a different sector: a cascading impact.

***2.3 Application of system dynamics to environmental domains***

While system dynamics has been applied primarily within the business or organisational management context (Burton et al., 2008), it has been applied to environmental and linked human-environmental systems since the 1970s, with the publication of ‘Limits to Growth’ (Meadows et al., 1974). Since that time, the use of System Dynamics in environmental management has grown exponentially, and is now used to address a wide-range of topics including air quality (Stave, 2002), park management (Kruse et al., 2004; Nicolson et al., 2002), water issues (Collins et al., 2007; Costanza et al., 1998; Moxey and White, 1998; Tidwell et al., 2004; Tidwell and Brink, 2008; van den Belt, 2004; van Eeten, 2001), sustainability (Williams et al., 2017), and soils studies (Inam et al., 2015).

There is limited system dynamics or critical systems thinking literature that has focused on the impacts or implications of climate change (Ison, 2010). Some examples include impacts on energy constraints and policy (Ansell and Cayzer, 2018), water resources (Winz et al., 2009) and water demand (Gastelum et al., 2018; Givens et al., 2018), food security (Guma et al., 2018), heat-related health impacts, and early thinking on resilient architectural design (Weisz, 2018). The focus is on parts of the system, using numerical modelling, and principally on biophysical or physical science subsystems. This leaves social systems largely unexamined in system dynamics framing, both by themselves or connected to the biophysical and physical world (Ison, 2010).

Conceptually however, systems thinking is well suited to providing a basis for understanding cascading systems, with its focus on critical thresholds in natural, built, and human systems, and interactions and feedback loops (Folke et al., 2010; Werners et al., 2013). Gaining insight into the scope of interconnectivity between internal and external stressors and sectors using critical systems thinking to describe cascading impacts and their implications, can support adaptation planning, helping to avoid maladaptation, and reducing the likelihood of negative cascades across the economy (Cash et al., 2006; Wilbanks and Kates, 2010; Yletyinen et al., 2019). A better understanding of the complexity of interacting and interconnected impacts can also help stakeholders’ to conceptualise the nature of climate change impacts, and thus facilitate the development of linked-up approaches to adaptation planning that consider upstream and downstream decision implications (Eakin et al., 2009; Fleming et al., 2014).

Finally, it should be noted that the application of System Dynamics exists along a spectrum of complexity; from conceptual System maps (also known as Causal Loop Diagrams or CLDs), to Stock and Flow Diagrams; to computer simulation modelling (based on the stock and flow formulation). The examples of System Dynamics tools shown in Figure 4 are used to highlight the potential for the same system – and its interrelationships - to be represented in a number of different ways, depending on the users’ needs, aims and objectives. Our methodology uses causal loop diagrams to characterise the cascading impacts of climate change for a particular system, however we encourage others to advance the theory and practice of cascades research using this, or other approaches.


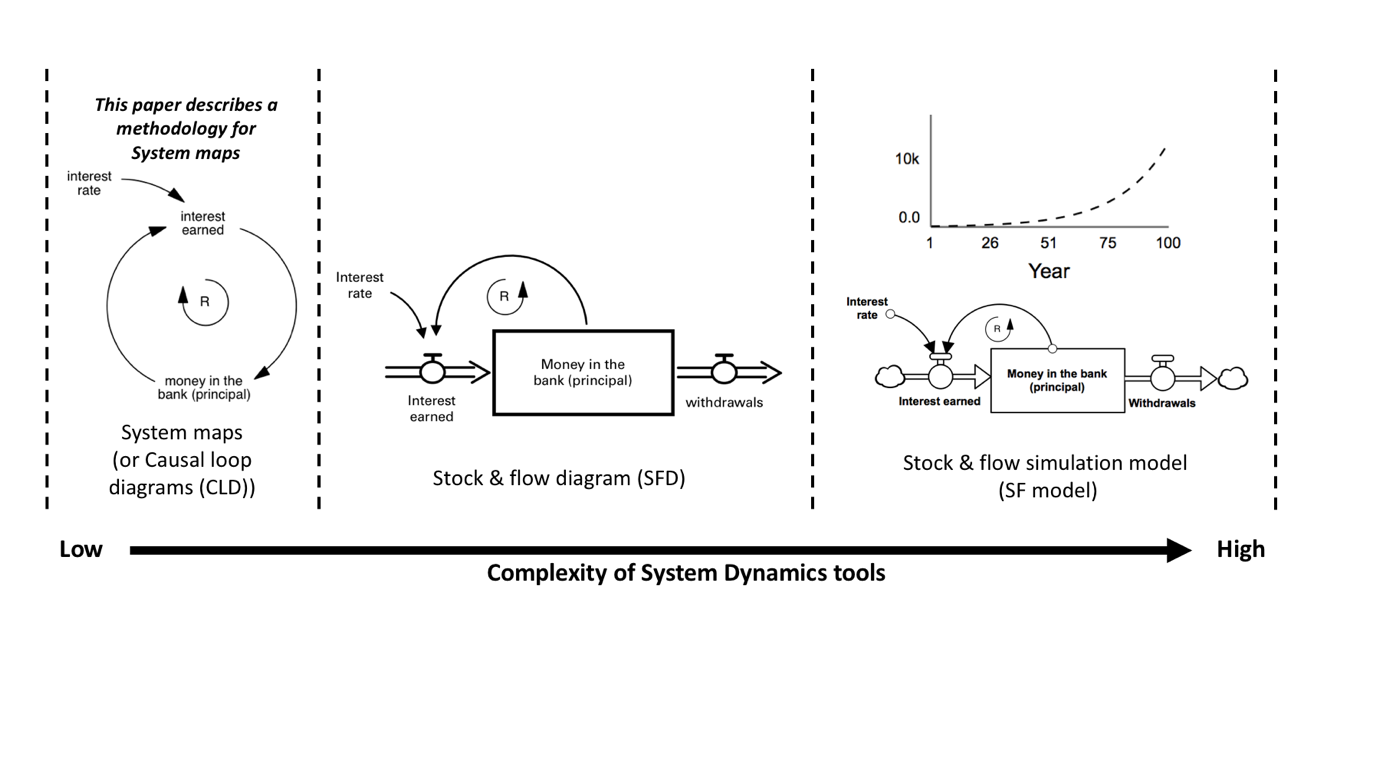


**Figure 4: System Dynamics tools exist on a spectrum of increasing complexity**

Abson, D.J., Fischer, J., Leventon, J., Newig, J., Schomerus, T., Vilsmaier, U., Wehrden, H. von, Abernethy, P., Ives, C.D., Jager, N.W., Lang, D.J., 2017. Leverage points for sustainability transformation. Ambio 46, 30–39. https://doi.org/10.1007/s13280-016-0800-y

Agrawal, A., Perrin, N., Chhatre, A., Benson, C.S., Kononen, M., 2013. Climate policy processes, local institutions, and adaptation actions: mechanisms of translation and influence. Wiley Interdisciplinary Reviews: Climate Change 4, 72–72. https://doi.org/10.1002/wcc.203

Ansell, T., Cayzer, S., 2018. Limits to growth redux: A system dynamics model for assessing energy and climate change constraints to global growth. Energy Policy 120, 514–525. https://doi.org/10.1016/j.enpol.2018.05.053

Barnes, M., Bodin, Ö., Guerrero, A., McAllister, R., Alexander, S., Robins, G., 2017. The social structural foundations of adaptation and transformation in social–ecological systems. Ecology and Society 22. https://doi.org/10.5751/ES-09769-220416

Bollinger, L.A., Bogmans, C.W.J., Chappin, E.J.L., Dijkema, G.P.J., Huibregtse, J.N., Maas, N., Schenk, T., Snelder, M., Thienen, P. van, Wit, S. de, Wols, B., Tavasszy, L.A., 2014. Climate adaptation of interconnected infrastructures: a framework for supporting governance. Reg Environ Change 14, 919–931. https://doi.org/10.1007/s10113-013-0428-4

Burton, Rob.J.F., Kuczera, C., Schwarz, G., 2008. Exploring Farmers’ Cultural Resistance to Voluntary Agri-environmental Schemes. Sociologia Ruralis 48, 16–37. https://doi.org/10.1111/j.1467-9523.2008.00452.x

Cash, D., Adger, W.N., Berkes, F., Garden, P., Lebel, L., Olsson, P., Pritchard, L., Young, O., 2006. Scale and Cross-Scale Dynamics: Governance and Information in a Multilevel World. Ecology and Society 11. https://doi.org/10.5751/ES-01759-110208

Collins, K., Blackmore, C., Morris, D., Watson, D., 2007. A systemic approach to managing multiple perspectives and stakeholding in water catchments: some findings from three UK case studies. Environmental Science & Policy 10, 564–574. https://doi.org/10.1016/j.envsci.2006.12.005

Costanza, R., Andrade, F., Antunes, P., van den Belt, M., Boersma, D., Boesch, D.F., Catarino, F., Hanna, S., Limburg, K., Low, B., Molitor, M., Pereira, J.G., Rayner, S., Santos, R., Wilson, J., Young, M., 1998. Principles for Sustainable Governance of the Oceans. Science 281, 198–199. https://doi.org/10.1126/science.281.5374.198

Dentoni, D., Bitzer, V., Schouten, G., 2018. Harnessing Wicked Problems in Multi-stakeholder Partnerships. J Bus Ethics 150, 333–356. https://doi.org/10.1007/s10551-018-3858-6

Eakin, H., Winkels, A., Sendzimir, J., 2009. Nested vulnerability: exploring cross-scale linkages and vulnerability teleconnections in Mexican and Vietnamese coffee systems. Environmental Science & Policy 12, 398–412. https://doi.org/10.1016/j.envsci.2008.09.003

Fischer, J., Riechers, M., 2019. A leverage points perspective on sustainability. People Nat 1, 115–120. https://doi.org/10.1002/pan3.13

Fleming, A., Hobday, A.J., Farmery, A., van Putten, E.I., Pecl, G.T., Green, B.S., Lim-Camacho, L., 2014. Climate change risks and adaptation options across Australian seafood supply chains – A preliminary assessment. Climate Risk Management 1, 39–50. https://doi.org/10.1016/j.crm.2013.12.003

Folke, C., Carpenter, S.R., Walker, B., Scheffer, M., Chapin, T., Rockström, J., 2010. Resilience Thinking: Integrating Resilience, Adaptability, and Transformability. Ecology and Society 15, 20.

Forrester, J.W., 1968. Principles of systems. Wright-Allen Press Inc, Cambridge, Mass.

Frame, B., 2008. ‘Wicked’, ‘messy’, and ‘clumsy’: long-term frameworks for sustainability. Environment and Planning C: Government and Policy 26, 1113 – 1128. https://doi.org/10.1068/c0790s

Galaz, V., Moberg, F., Olsson, E.-K., Paglia, E., Parker, C., 2011. Institutional and Political Leadership Dimensions of Cascading Ecological Crises. Public Administration 89, 361–380. https://doi.org/10.1111/j.1467-9299.2010.01883.x

Gastelum, J.R., Krishnamurthy, G., Ochoa, N., Sibbett, S., Armstrong, M., Kalaria, P., 2018. The Use of System Dynamics Model to Enhance Integrated Resources Planning Implementation. Water Resour Manage 32, 2247–2260. https://doi.org/10.1007/s11269-018-1926-4

Givens, J.E., Padowski, J., Guzman, C.D., Malek, K., Witinok-Huber, R., Cosens, B., Briscoe, M., Boll, J., Adam, J., 2018. Incorporating Social System Dynamics in the Columbia River Basin: Food-Energy-Water Resilience and Sustainability Modeling in the Yakima River Basin. Front. Environ. Sci. 6. https://doi.org/10.3389/fenvs.2018.00104

Guma, I.P., Rwashana, A.S., Oyo, B., 2018. Food Security Policy Analysis Using System Dynamics: The Case of Uganda. IJITSA 11, 72–90. https://doi.org/10.4018/IJITSA.2018010104

Holling, C.S., 2001. Understanding the Complexity of Economic, Ecological, and Social Systems. Ecosystems 4, 390–405. https://doi.org/10.1007/s10021-001-0101-5

Inam, A., Adamowski, J., Halbe, J., Prasher, S., 2015. Using causal loop diagrams for the initialization of stakeholder engagement in soil salinity management in agricultural watersheds in developing countries: A case study in the Rechna Doab watershed, Pakistan. Journal of Environmental Management 152, 251–267. https://doi.org/10.1016/j.jenvman.2015.01.052

Ison, R.L., 2010. Systems Practice: How to Act in a Climate Change World. Berlin, Springer.

Keys, P.W., Galaz, V., Dyer, M., Matthews, N., Folke, C., Nyström, M., Cornell, S.E., 2019. Anthropocene risk. Nat Sustain 2, 667–673. https://doi.org/10.1038/s41893-019-0327-x

Kruse, J.A., White, R.G., Epstein, H.E., Archie, B., Berman, M., Braund, S.R., Chapin, F.S., Charlie, J., Daniel, C.J., Eamer, J., Flanders, N., Griffith, B., Haley, S., Huskey, L., Joseph, B., Klein, D.R., Kofinas, G.P., Martin, S.M., Murphy, S.M., Nebesky, W., Nicolson, C., Russell, D.E., Tetlichi, J., Tussing, A., Walker, M.D., Young, O.R., 2004. Modeling Sustainability of Arctic Communities: An Interdisciplinary Collaboration of Researchers and Local Knowledge Holders. Ecosystems 7, 815–828. https://doi.org/10.1007/s10021-004-0008-z

Liu, J., Hull, V., Batistella, M., DeFries, R., Dietz, T., Fu, F., Hertel, T., Izaurralde, R.C., Lambin, E., Li, S., Martinelli, L., McConnell, W., Moran, E., Naylor, R., Ouyang, Z., Polenske, K., Reenberg, A., de Miranda Rocha, G., Simmons, C., Verburg, P., Vitousek, P., Zhang, F., Zhu, C., 2013. Framing Sustainability in a Telecoupled World. Ecology and Society 18. https://doi.org/10.5751/ES-05873-180226

Meadows, D., 2010. Leverage Points: Places to Intervene in a System. Solutions 1, 41–49.

Moser, S.C., Jeffress Williams, S., Boesch, D.F., 2012. Wicked Challenges at Land’s End: Managing Coastal Vulnerability Under Climate Change. Annual Review of Environment and Resources 37, 51–78. https://doi.org/10.1146/annurev-environ-021611-135158

Moxey, A., White, B., 1998. NELUP: Some Reflections on Undertaking and Reporting InterdisciplinaryRiver Catchment Modelling. Journal of Environmental Planning and Management 41, 397–402. https://doi.org/10.1080/09640569811650

Nicolson, C.R., Starfield, A.M., Kofinas, G.P., Kruse, J.A., 2002. Ten Heuristics for Interdisciplinary Modeling Projects. Ecosystems 5, 376–384. https://doi.org/10.1007/s10021-001-0081-5

Ross, H., Shaw, S., Rissik, D., Cliffe, N., Chapman, S., Hounsell, V., Udy, J., Trinh, N.T., Schoeman, J., 2015. A participatory systems approach to understanding climate adaptation needs. Climatic Change 129, 27–42. https://doi.org/10.1007/s10584-014-1318-6

Senge, P.M., 2006. The Fifth Discipline: The Art and Practice of the Learning Organization, 2nd ed. Doubleday, New York, NY.

Stave, K.A., 2002. Using system dynamics to improve public participation in environmental decisions. System Dynamics Review 18, 139–167. https://doi.org/10.1002/sdr.237

Sterman, J.D., 2000. Business dynamics: Systems thinking and modelling for a complex world. McGraw-Hill, New York, NY.

Tidwell, V.C., Brink, C.V.D., 2008. Cooperative Modeling: Linking Science, Communication, and Ground Water Planning. Groundwater 46, 174–182. https://doi.org/10.1111/j.1745-6584.2007.00394.x

Tidwell, V.C., Passell, H.D., Conrad, S.H., Thomas, R.P., 2004. System dynamics modeling for community-based water planning: Application to the Middle Rio Grande. Aquat. Sci. 66, 357–372. https://doi.org/10.1007/s00027-004-0722-9

van den Belt, M., 2004. Mediated Modeling: A System Dynamics Approach To Environmental Consensus Building. Island Press.

van Eeten, M.J.G., 2001. Recasting Intractable Policy Issues: The Wider Implications of The Netherlands Civil Aviation Controversy. Journal of Policy Analysis and Management 20, 391–414. https://doi.org/10.1002/pam.1000

Vennix, J.A.M., Akkermans, H.A., Rouwette, E.A.J.A., 1996. Group model-building to facilitate organizational change: an exploratory study. System Dynamics Review 12, 39–58. https://doi.org/10.1002/(SICI)1099-1727(199621)12:1<39::AID-SDR94>3.0.CO;2-K

Weisz, C., 2018. Resilient Design: ‘Systems Thinking’ as a Response to Climate Change. Architectural Design 88, 24–31. https://doi.org/10.1002/ad.2255

Werners, S.E., Pfenninger, S., van Slobbe, E., Haasnoot, M., Kwakkel, J.H., Swart, R.J., 2013. Thresholds, tipping and turning points for sustainability under climate change. Current Opinion in Environmental Sustainability. https://doi.org/10.1016/j.cosust.2013.06.005

Wilbanks, T.J., Kates, R.W., 2010. Beyond Adapting to Climate Change: Embedding Adaptation in Responses to Multiple Threats and Stresses. Annals of the Association of American Geographers 100, 719–728. https://doi.org/10.1080/00045608.2010.500200

Williams, A., Kennedy, S., Philipp, F., Whiteman, G., 2017. Systems thinking: A review of sustainability management research. Journal of Cleaner Production 148, 866–881. https://doi.org/10.1016/j.jclepro.2017.02.002

Winz, I., Brierley, G., Trowsdale, S., 2009. The Use of System Dynamics Simulation in Water Resources Management. Water Resour Manage 23, 1301–1323. https://doi.org/10.1007/s11269-008-9328-7

Yletyinen, J., Brown, P., Pech, R., Hodges, D., Hulme, P.E., Malcolm, T.F., Maseyk, F.J.F., Peltzer, D.A., Perry, G.L.W., Richardson, S.J., Smaill, S.J., Stanley, M.C., Todd, J.H., Walsh, P.J., Wright, W., Tylianakis, J.M., 2019. Understanding and Managing Social–Ecological Tipping Points in Primary Industries. BioScience 69, 335–347. https://doi.org/10.1093/biosci/biz031
